# Predictors of Facial Pain and Headache Associated With Cryotherapy Ablation of the Posterior Nasal Nerve for the Treatment of Chronic Rhinitis

**DOI:** 10.7759/cureus.61749

**Published:** 2024-06-05

**Authors:** Samuel E Razmi, Aatin K Dhanda, Jason Shenoi, Faizaan Khan, Masayoshi Takashima, Omar G Ahmed

**Affiliations:** 1 School of Engineering Medicine, Texas A&M (Agricultural and Mechanical) College of Medicine, Houston, USA; 2 Department of Otolaryngology - Head and Neck Surgery, Houston Methodist Hospital, Houston, USA

**Keywords:** chronic rhinitis, facial pain, headache, cryotherapy ablation, posterior nasal nerve

## Abstract

Objective: Cryotherapy ablation of the posterior nasal nerve (PNN) for treatment of patients with refractory chronic rhinitis (CR) is associated with postoperative facial pain and headache. This study sought to understand factors that may contribute to the development of this adverse effect.

Methods: Patients undergoing PNN cryotherapy ablation for refractory CR at a single institution from January 2018 to August 2023 were included. Demographics and clinical characteristics were collected via chart review and interview. Student’s T-test and Chi-square tests were used to assess the significance of quantitative and categorical data, respectively (alpha = 0.05).

Results: Forty-eight patients underwent cryotherapy ablation. Twenty-eight patients (58%) reported having facial pain and headache (adverse effect group) immediately post-procedurally. The average age of the adverse effect group was 54.9 years (SD: 17.8 years) which was significantly lower (p=0.002) than the asymptomatic group (69.7 years, SD: 8.7 years). Female patients were significantly more likely to experience this adverse event than males (p=0.04). Moreover, Caucasian females were significantly more likely to experience this adverse effect when compared to all patients experiencing the adverse effect (n=15, p=0.04). Previous diagnosis of migraine disorder was more common in the adverse effect group (28%) compared to the asymptomatic group (15%) but not statistically significant (p=0.26). Previous migraine, trigeminal neuralgia, or headache disorder diagnoses were not significantly correlated with adverse effect prevalence (p = 0.26, 0.24, 0.15, respectively).

Conclusion: Given the relative immediacy and severity of this adverse effect, physicians should strongly consider these factors when counseling and selecting certain patient groups for this procedure.

## Introduction

Chronic rhinitis (CR) is a prevalent condition affecting over 60 million people per year; however, 10-22% of these patients are refractory to medical treatment [1]. For these patients, treatments such as cryotherapy ablation of the posterior nasal nerve (PNN) have emerged as an effective option for patients with refractory CR, showing sustained improvement in nasal congestion and rhinorrhea with a relatively acceptable safety profile [1,2].

However, few studies specifically evaluate post-procedure adverse events. The procedure is often associated with postoperative facial pain and headache, colloquially described as an “ice cream headache" [3]. It is unclear why this occurs, but it is hypothesized that cryotherapy ablation in the posterior middle meatus, near the pterygopalatine fossa, may activate the trigeminal pathway. We sought to understand factors that may contribute to the development of this dreaded adverse effect.

## Materials and methods

As a part of the primary study, patients undergoing PNN cryotherapy ablation for refractory CR at a single institution from January 2018 to August 2023 were included. Patients were included if they were 18 years an older with confirmed diagnosis chronic rhinitis refractory to traditional therapy who had undergone PNN cryotherapy ablation. Patients who had no recollection of the procedure or lost to follow up were not included in the study. Prior to the procedure, 6% tetracaine soaked pledgets were placed in the middle meatus for 10 minutes followed by 1 cc injection of 1% lidocaine with epinephrine (1:100,000) into the sphenopalatine foramen region bilaterally. Demographics and clinical characteristics were collected via chart review and interview. Patient interviews were standardized to avoid biasing responses. Care was taken to use appropriate question clarifications and probes (Table 1).

**Table 1:** Standardized questions asked in interview format to obtain patient information. IRB: Institutional Review Board.

Category	Questions
Introduction	Hello, may I speak with [Patient]?
	This is [Name], I’m a [Role] affiliated with Houston Methodist Hospital.
	We are interested in learning more about a procedure you may have undergone at Houston Methodist with Dr. Omar Ahmed or Dr. Masayoshi Takashima.
	This is an IRB-approved study, meaning your data is protected and confidential. Are you interested in participating?
Data acquisition	Do you remember having a procedure for your rhinitis or nasal symptoms?
	Do you remember having any side effects afterward?
	If so, [length of side effect, severity of a scale of 1-10, resolving factors, etc.]
	Now, we are going to obtain some data on your current post-procedure symptoms, if you have any. For each of the symptoms, you will rate them on a scale of 0-3. 0 = no symptoms. 1 = mild symptoms, aware but not troubled. 2 = moderate, troublesome but not interfering with normal daily activities or sleep 3 = severe, interfering with normal daily activities or sleep. [Obtained for Rhinorrhea, Sneezing, Itching, and Congestion.]
	Do you feel like the procedure improved your symptoms? If so, how much on a scale from 1-100%?
Wrap-up	Do you have any questions for me?
	If you think of any further questions, please feel free to email me at [Email] or call/text me at [Phone Number]. Thank you and have a nice day.

Given the symptoms of facial pain and headache often overlapped, these adverse events were grouped together. Adverse effect pain was assessed via a verbal pain scale (1-10, mild = 1-3, moderate = 4-6, severe = 7-10). Post-procedural total nasal symptom score (TNSS) was obtained during patient interviews. Student’s T-test and Chi-square tests were used to assess the significance of quantitative and categorical data, respectively (alpha = 0.05).

## Results

Forty-eight patients underwent cryotherapy ablation. Twenty-eight patients (58%) reported having facial pain and headache (adverse effect group) immediately post-procedurally with a mean duration of 65 minutes (SD: 43 minutes, range: 5-180 minutes) with an average pain rating of 8.01 (SD: 1.78, 10 = worst pain). Seventy-one percent of all patients reported clinically meaningful improvement of chronic rhinitis symptoms post-procedurally, defined as a 30% or greater improvement in symptomology. There was no statistically significant difference between the adverse effect group and asymptomatic group for post-procedural symptom relief (p=0.37) (Table 2).

**Table 2:** Data demonstrating results of various statistical tests run on several major variables. TNSS: Total Nasal Symptom Score; M: Meter; Hx: History.

Category	Subcategory	Adverse effect	Asymptomatic	P-value
Age (years)		54.9 ± 17.8	69.7 ± 8.7	0.002
Postoperative TNSS		3.5 ± 2.0	2.45 ± 2.49	0.11
Height (M)		1.7 ± 0.1	1.7 ± 0.1	0.13
Body Mass Index (BMI)		27.8 ± 5.9	29.37 ± 5.7	0.43
Gender				
	Female	19	6	0.04
	Male	9	14	
Race				
	Caucasian	23	15	0.62
	Black	3	2	
	Hispanic	2	2	
Symptom relief				
	Positive	19	15	0.37
	Negative	9	5	
Hx of reflux				
	Positive	5	6	0.29
	Negative	23	14	
Hx of trimgenal neuralgia				
	Positive	0	1	0.24
	Negative	28	19	
Hx of migraine				
	Positive	8	3	0.26
	Negative	20	17	
Hx of headache disorder				
	Positive	0	2	0.15
	Negative	28	18	

The average age of the adverse effect group was 54.9 years (SD: 17.8 years) which was significantly lower (p=0.002) than the asymptomatic group (69.7 years, SD: 8.7 years). Female patients were significantly more likely to experience this adverse event than males (p=0.04). Moreover, Caucasian females were significantly more likely to experiences this adverse effect when compared to all patients experiencing the adverse effect (n=15, p=0.04). There was no statistical difference in the overall postoperative TNSS score between the adverse effect and non-asymptomatic group (p=0.11). Body mass index and height were not found to correlate with adverse effect prevalence. Previous diagnosis of migraine disorder was more common in the adverse effect group (28%) compared to the asymptomatic group (15%) but not statistically significant (p=0.26). Previous migraine, trigeminal neuralgia, or headache disorder diagnosis were not significantly correlated with adverse effect prevalence (p = 0.26, 0.24, 0.15, respectively).

## Discussion

This study investigated patient factors associated with postoperative facial pain and headache following cryotherapy ablation of the PNN for the treatment of refractory chronic rhinitis.

Postoperative facial pain and headache were reported more frequently in our cohort (58%) compared to previous literature which reports a 7.5-40% rate for headaches/facial pain in variable sample sizes [1,3-5]. Although facial pain and headache can sometimes be considered separate symptoms, our study combined these symptoms given the difficulty many of our patients had differentiating between the two. Overall, most patients still reported improvement of chronic rhinitis symptoms from the procedure postoperatively and there was no difference in symptom relief between both groups, demonstrating that the overall efficacy of the procedure remains substantial despite this adverse effect. 

While no differences in procedural efficacy were noted, understanding the incidence and predictive factors for the development of this adverse event can help clinicians counsel patients most likely to be at risk. This study found younger (average age 55 vs 69, p=.002), females compared to males (76% vs 39% p=.04), and females of the Caucasian race (p=.04) were more likely to develop postoperative facial pain and headache. Caucasian females have similarly been noted to have an increased risk of migraines [6]. Stewart et al. found that gender and race-related differences in genetic vulnerability to migraines could be a potential explanation for this trend [6]. In our study, previous diagnoses of migraines were found to be more prevalent in the adverse effect group but did not reach statistical significance. There was no significant difference in the adverse effect group for patients with a history of trigeminal neuralgia and other headache disorders. Furthermore, differences in factors such as socioeconomic status, diet, and even emotional state at the time of procedure must also be considered. The underlying mechanisms of why gender, ethnicity, and age influence perceived pain are uncertain, but some studies support inherent differences between groups [7,8]. Thought should also be given to which groups of people are most likely to report pain as well. For example, race may affect the perception of pain and the desire to seek treatment [9]. Given the relative immediacy and severity of this adverse effect, physicians should strongly consider these factors when counseling and selecting certain patient groups for this procedure. Consideration should also be given to using preoperative analgesia as its use may considerably reduce this adverse effect [10].

This study is limited to a single institution retrospective analysis. Patients’ recall bias may affect their reporting of symptoms and adverse of events. Future prospective studies should include multicenter cohorts with larger datasets to validate and extend these findings.

## Conclusions

This study investigated factors associated with postoperative facial pain and headache following cryotherapy ablation of the posterior nasal nerve for refractory chronic rhinitis. Younger age, female gender, and Caucasian females were found to be associated with a higher risk of developing this adverse effect. Understanding these risk factors can help clinicians better counsel patients who are more likely to experience this adverse effect. Future prospective studies with larger, multicenter cohorts are needed to validate these findings and improve patient counseling and treatment planning.

## References

[1] Ow RA, O'Malley EM, Han JK, Lam KK, Yen DM (2021). Cryosurgical ablation for treatment of rhinitis: two-year results of a prospective multicenter study. Laryngoscope.

[2] (2023). ARS position statement: posterior nasal nerve ablation. https://www.american-rhinologic.org/index.php?option=com_content&view=article&id=478:posterior-nasal-nerve-ablation&catid=26:position-statements&Itemid=197.

[3] Chang MT, Song S, Hwang PH (2020). Cryosurgical ablation for treatment of rhinitis: a prospective multicenter study. Laryngoscope.

[4] M Yen D, B Conley D, O'Malley EM, Byerly TA, Johnson J (2020). Multiple site cryoablation treatment of the posterior nasal nerve for treatment of chronic rhinitis: an observational feasibility study. Allergy Rhinol (Providence).

[5] Yoo F, Kuan EC, Batra PS, Chan CK, Tajudeen BA, Craig JR (2020). Predictors of rhinorrhea response after posterior nasal nerve cryoablation for chronic rhinitis. Int Forum Allergy Rhinol.

[6] Stewart WF, Lipton RB, Liberman J (1996). Variation in migraine prevalence by race. Neurology.

[7] Sheffield D, Biles PL, Orom H, Maixner W, Sheps DS (2000). Race and sex differences in cutaneous pain perception. Psychosom Med.

[8] Wandner LD, Scipio CD, Hirsh AT, Torres CA, Robinson ME (2012). The perception of pain in others: how gender, race, and age influence pain expectations. J Pain.

[9] Wyatt R (2013). Pain and ethnicity. Virtual Mentor.

[10] Steele TO, Hoshal SG, Kim M (2020). A preliminary report on the effect of gabapentin pretreatment on periprocedural pain during in-office posterior nasal nerve cryoablation. Int Forum Allergy Rhinol.

